# Examination of the APOBEC3 Barrier to Cross Species Transmission of Primate Lentiviruses

**DOI:** 10.3390/v13061084

**Published:** 2021-06-07

**Authors:** Amit Gaba, Ben Flath, Linda Chelico

**Affiliations:** Department of Biochemistry, Microbiology, and Immunology, University of Saskatchewan, Saskatoon, SA S7H 0E5, Canada; amg340@usak.ca (A.G.); bef206@usak.ca (B.F.)

**Keywords:** immunodeficiency virus, APOBEC3, restriction factor, Vif, protein-protein interactions, cross-species infection, virus transmission

## Abstract

The transmission of viruses from animal hosts into humans have led to the emergence of several diseases. Usually these cross-species transmissions are blocked by host restriction factors, which are proteins that can block virus replication at a specific step. In the natural virus host, the restriction factor activity is usually suppressed by a viral antagonist protein, but this is not the case for restriction factors from an unnatural host. However, due to ongoing viral evolution, sometimes the viral antagonist can evolve to suppress restriction factors in a new host, enabling cross-species transmission. Here we examine the classical case of this paradigm by reviewing research on APOBEC3 restriction factors and how they can suppress human immunodeficiency virus (HIV) and simian immunodeficiency virus (SIV). APOBEC3 enzymes are single-stranded DNA cytidine deaminases that can induce mutagenesis of proviral DNA by catalyzing the conversion of cytidine to promutagenic uridine on single-stranded viral (−)DNA if they escape the HIV/SIV antagonist protein, Vif. APOBEC3 degradation is induced by Vif through the proteasome pathway. SIV has been transmitted between Old World Monkeys and to hominids. Here we examine the adaptations that enabled such events and the ongoing impact of the APOBEC3-Vif interface on HIV in humans.

## 1. Introduction

Spillover of viruses from animal hosts have led to the emergence of several human diseases including COVID-19, severe acute respiratory syndrome (SARS), Nipah, Ebola, influenza and acquired immunodeficiency syndrome (AIDS) [1,2,3,4]. Several factors including the nature of contact between the animal host and humans, host restriction factors and viral factors determine the establishment of a new virus and its spread within the human population [1,2,3,4]. To establish replication in a host, a virus needs to overcome several restrictions posed by the host at various stages of the virus life cycle including entry of the virus in appropriate cells, trafficking within the cell, replication, assembly, and release. The host factors that cause these restrictions and make the host cell resistant to viral infection are referred to as cellular restriction factors [5]. To overcome these multiple barriers presented by the host, the virus must make corresponding changes in its genome [6]. Thus, these cellular restriction factors are always evolving under strong positive selection pressure to acquire adaptations that circumvent suppression by viral pathogens. 

This positive selection of restriction factors leads to differences in functionality, even in similar species, resulting in the ability of these cellular restriction factors to also act as barriers to the cross species transmission of viruses [7]. To successfully overcome these barriers and infect a new host, the virus needs to make adaptive changes in its genes that can counteract the new host’s restriction factor. To prove that a particular host factor can act as cross species transmission barrier it is imperative to identify changes in the virus correlating to host switching events and demonstrating a gain of function to counteract the host restriction factor in the new host. 

AIDS, which is caused by the human immunodeficiency virus (HIV), is a classic example of spillover of an animal virus into humans. HIV and related immunodeficiency viruses are lentiviruses, a genus of retroviruses where infection is characterized by long incubation periods between infection and onset of disease. The study of the emergence of AIDS also exemplifies how a cellular restriction factor can act as a cross species transmission barrier and how a virus adapts to overcome this barrier. This review examines cross species transmission of the simian immunodeficiency virus (SIV) between primates, with specific focus on the APOBEC3 family of cellular restriction factors that act as single-stranded (ss) DNA cytosine deaminases. 

## 2. Origin of the Immunodeficiency Virus in Humans

The world first became aware of AIDS in the early 1980s, although it had become well established in the human population within the Belgian colonized region of the Congo in the early 1900s [8]. Colonization practices enhanced the interaction of people with primates, led to the reuse of syringes in population wide medical programs, and increased the number of clients for each sex worker, which fueled spread and adaptation of HIV [8,9,10]. Two lentiviruses—human immunodeficiency viruses Type 1 and Type 2 (HIV-1 and HIV-2)—can cause AIDS in humans. Ever since HIV was first discovered, the reason for its emergence and origin have been subject of intense study. Over the years, scientists have made significant progress in tracing the history of HIV, using advanced genetic and biochemical techniques. Many species of Old World Monkey (OWM) have been found to be infected with SIV. SIVs are present in more than 40 non-human primate species [11]. The only three primate species to transmit their viruses to humans are chimpanzees, gorillas and sooty mangabeys [1,12]. 

Each of the groups of HIV originated from a single species jump. There are four lineages within HIV-1, groups M, N, O, and P. Groups M and N have been shown to originate from crossover events from chimpanzee. Group P is a jump from gorillas, and the evidence is not clear whether Group O originated from a crossover event from a chimpanzee or gorilla [1,12]. Given their close genetic relationship, it is plausible that chimpanzees and gorillas, but not monkeys transmitted their viruses to humans. However, there is one remarkable exception. The SIV_smm_ of sooty mangabeys was transmitted to humans at least nine independent occasions giving rise to HIV-2 groups A-I [1,13]. At each of these cross-species transmission events, virus adaptation to host restriction factors was necessary for successful infection and transmission within the new host group. 

## 3. Lentiviral Restriction Factors and Their Viral Antagonists

Restriction factors are cell intrinsic immune proteins that can restrict the replication of a virus at a specific step of replication or infection [5]. These proteins can decrease virus replication significantly, usually have their expression responsive to the innate immune response, and are at most times, susceptible to a viral counteraction mechanism, which often is a protein–protein interaction between the host and virus proteins [5]. Due to this combat at the protein level, the restriction factors show signs of positive selection indicating that rapid evolution is occurring, usually at the protein–protein interface between the virus and the host. This ensures, that in time, the restriction factor will overcome, at least partially, the viral antagonist, until further evolution of the viral antagonist protein takes place [5,6].

Several cellular restriction factors have specific viral antagonists. Bone marrow stromal antigen 2 (BST-2) or tetherin is a cellular transmembrane protein that blocks viral release from cells, causing them to be endocytosed and degraded [14]. The action of tetherin is suppressed by Vpu or Nef in HIV-1 and Env (envelope glycoprotein) in HIV-2 and SIV [15]. Another type of membrane protein, serine incorporator protein 3 (SERINC3) and SERINC5 can inhibit viral fusion, but this action is suppressed by Nef or Env in HIV-1, HIV-2 and SIV [16,17]. The SAM and HD domain-containing protein 1 (SAMHD1) in dendritic and myeloid cells reduces the intracellular deoxynucleotide triphosphate pool through its triphosphohydrolase activity which prevents completion of reverse transcription, but is suppressed by Vpx which is produced from HIV-2 and SIV, but not HIV-1 [18,19]. The focus of this review, the apolipoprotein B mRNA-editing enzyme, catalytic polypeptide-like 3 (APOBEC3) family of enzymes are ssDNA cytosine deaminases that can induce mutation of proviral DNA, but are suppressed by Vif in HIV-1, HIV-2, and SIV [20]. Interestingly, all of these counteraction mechanisms by the virus hijack cellular degradation pathways, either through the lysosome or proteasome. However, the substrates for degradation are usually virus and host specific. For example, the Vif of SIV from an OWM can induce degradation of that monkey’s APOBEC3 enzymes, (e.g., African Green Monkey (AGM)), but not those of humans. Thus, the cross species barrier is formed. Other restriction factors without viral antagonists are part of the interferon response that greatly decreases HIV infection and is mediated by tripartite motif-containing protein 5α (TRIM5α) and myxovirus resistance B (MxB), among others [21]. TRIM5α destabilizes capsid uncoating and MxB inhibits nuclear import of the preintegration complex [22,23,24,25,26,27].

Among all these restriction factors, APOBEC3s are one of the most potent restriction factors that are known to restrict lentivirus replication. Their multifunctional restriction mechanism and the number of paralogs that can restrict lentiviruses is unique among the restriction factors. Sheehy et al. first reported identification of a protein called CEM-15, later called apolipoprotein B mRNA editing enzyme catalytic polypeptide–like 3G (APOBEC3G; A3G) as a novel cellular restriction factor that inhibits HIV-1 replication [28]. Shortly thereafter it was realized that there are seven A3 enzymes in humans that exist in a tandem cluster on Chromosome 22 and are named alphabetically (A3A, A3B, A3C, A3D, A3F, A3G, and A3H) [29,30]. Despite this featureless naming system, each A3 enzyme has unique properties that results in differing abilities to restrict the replication of viral pathogens, with their restriction of lentivirus and retrotransposon replication being most studied [31,32]. However, as will be described, only those A3 enzymes localized in the cytoplasm can restrict lentiviruses, whereas others that localize to the nucleus can restrict viruses with nuclear replication, such as DNA viruses [33]. APOBEC3s in both the nucleus and cytoplasm can restrict retrotransposons [34].

## 4. Overview of APOBEC3s

The A3 family of enzymes are all cytosine deaminases on ssDNA, although some can also deaminate RNA [31,35]. In primates, there are at least seven members in the A3 family [36]. Although initially given the letter names, there is an additional and more universal naming system based on their conserved zinc-dependent deaminase domain (ZDD) [37]. The characteristic feature of the A3 family is that they contain one (A3A, A3C, A3H) or two (A3B, A3D, A3F, A3G) copies of a ZDD, with the consensus sequence H-X-E-X_23-28_-P-C-X_2-4_-C [37]. The variation in the ZDD consensus sequence results in three distinct phylogenetic clusters termed Z1, Z2 or Z3 [37]. In humans, A3H represents the only Z3 domain [37]. Some double domain enzymes, such as A3G are a Z2-Z1 type, but others, such as A3D and A3F are Z2-Z2 [37]. For human A3 enzymes that have two ZDD, only the C-terminal domain (CTD) is catalytically active, although both coordinate Zn^2+^ [38]. The histidine and cysteines of the Z-domain coordinate the Zn^2+^ while the glutamate participates in deaminase activity by shuttling the proton that activates a water molecule for nucleophilic attack of the cytosine in the active domain [39,40]. A3 enzymes deaminate within a preferred di- or tri- nucleotide substrate motif. A3G preferentially deaminates 5’CCC or 5’CC (underlined primarily C deaminated) while A3D, A3F, A3H and A3C prefer a 5’TTC or 5’TC motif [41,42,43]. It has been reported that A3D can also deaminate 5’GC motif [44]. 

**Figure 1:**
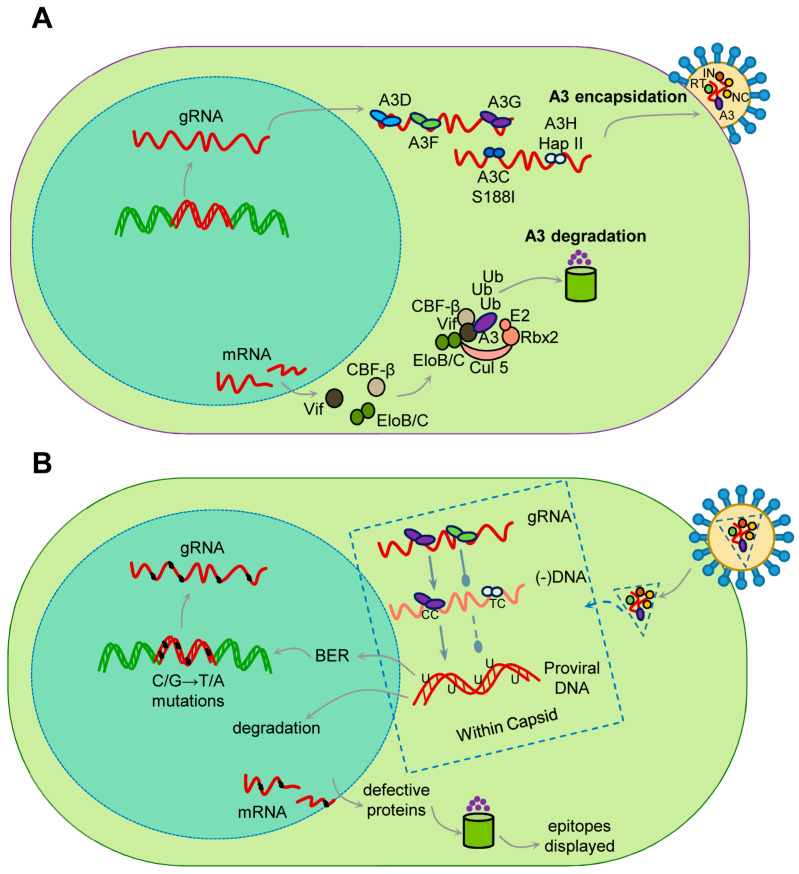
APOBEC3-mediated restriction of HIV-1 and Vif counteraction. (**A**) An HIV or SIV retrovirus integrates into the host genome as a double-stranded DNA provirus (red). This provirus is transcribed to produce genomic RNA (gRNA) and mRNA. The gRNA is exported to the cytoplasm where it is bound by viral Gag polyprotein to facilitate encapsidation (not shown). Additionally, in the cytoplasm are the APOBEC3 (A3) enzymes A3D, A3F, A3G, A3C S188I, and A3H Haplotypes II, V, and VII. The A3 enzymes bind the gRNA and become encapsidated if they escape Vif mediated degradation. There are also the viral proteins reverse transcriptase (RT), nucleocapsid (NC), integrase (IN) among others, with the encapsidated A3. The viral mRNA produces the protein Vif later in the replication cycle and with the highest Vif levels correlating with virus assembly, to purge the cytoplasm of A3 enzymes. However, Vif-mediated degradation is often incomplete. Vif binds CBF-β and Elongin C (EloC) which stabilizes Vif and enables nucleation of the Cullin 5 E3 ubiquitin ligase complex which ubiqutinates A3 enzymes and induces their degradation through the proteasome pathway. (**B**) A3 enzymes that escape this fate travel to the next target cell of infection within the viral capsid. The capsid stays intact during reverse transcription and is drawn as an expanded view in a box. Here the viral reverse transcriptase copies the gRNA to (−)DNA. This forms a RNA/DNA hybrid (not shown) that is converted to ssDNA by the endonuclease action of the RNaseH domain of reverse transcriptase. During this short time before (+)DNA synthesis starts, A3 enzymes access ssDNA and inhibit reverse transcriptase DNA synthesis from the RNA (round head arrow) or DNA (hatched round head arrow) template, but the blockage is more efficient from the RNA template. Primarily the A3 enzymes deaminate cytosine (C)→ uracil (U) in a 5’CC or 5’TC context. Copying a U in DNA leads to an adenine (A) being inserted on the (+)DNA strand. This causes G→A mutations in the coding strand sense. The uracils can be repaired by host base excision repair (BER) and the proviral DNA can integrate into the host genome. Alternatively, excessive uracils can lead to DNA repair mediated degradation, which destroys the proviral DNA. Integrated but mutated proviral DNA can remain transcriptionally active but does not produce virions. The mRNA can, however, be translated. The defective proteins may be misfolded or truncated leading to their degradation in the proteasome which promotes display of their epitopes on the surface of infected cells and promotes immune recognition.

When a lentivirus, such as HIV-1, infects a CD4+ T cell where A3s are expressed, the A3s in the cytoplasm are able to become encapsidated into newly formed virions (Figure 1). In humans, A3G, A3F, A3D, A3H (Haplotypes II, V, and VII), and A3C variant S188I are able to restrict HIV-1 replication to varying degrees [28,42,43,44,45,46,47,48]. A3H occurs in primates as several different haplotypes with differing cellular stabilities based on their propensity to become ubiquitinated and different cellular localizations [49,50]. The haplotypes that restrict lentiviruses are stable in cells and localized to the cytoplasm [45,49,50,51,52]. For A3C, the common form does not restrict HIV, although it is encapsidated, but the S188I variant present in 10% of people of African descent, and is able to restrict HIV due to an acquired ability to dimerize, which increases its restriction ability [48,53]. These A3s are able to bind viral genomic RNA and/or HIV-1 Gag, which enables their encapsidation [54,55,56]. Other A3s such as A3A and A3B do not restrict HIV-1 in CD4+ T cells. A3A is primarily expressed in cells of the monocyte lineage and is not encapsidated into virions due to a low binding affinity for RNA [57,58,59,60]. A3B has a nuclear localization signal and is not in the correct cellular localization for encapsidation [61,62]. When these virions infect a new cell, the encapsidated A3 can restrict viral replication by binding to the genomic RNA to physically inhibit reverse transcriptase during synthesis of (−)DNA from the RNA genome or the (+)DNA to form the double-stranded DNA provirus (Figure 1) [63,64,65,66]. However, the more dominant mode of restriction is by enzymatically deaminating cytosines on single-stranded (−)DNA that forms multiple uracils, which are promutagenic in DNA since they template for the addition of adenine, leading to mutation of the viral genome when uracil containing (−)DNA is used as a template for synthesizing (+)DNA [41,67,68,69]. These events occur in the viral capsid which is transported on microtubules to the nuclear pore and imported into the nucleus where reverse transcription and capsid disassembly completes [70,71,72,73,74]. The mutagenesis is facilitated by the host DNA repair enzymes that remove uracil and insert a thymine opposite the (+)DNA adenine. Hypermutation occurs when Vif is absent, and results in at least 10 mutations/kb on average in the ~10 kb HIV-1 genome (Figure 1) [75]. When Vif is present, the mutations decrease to ~1.8 mutations/kb [75]. These high mutation rates are due to the efficient processivity of A3 enzymes that enables a fast search for target cytosine containing motifs for deamination, before the (−)DNA becomes double stranded [32]. The mutated proviral DNA can become integrated into the host genome and remain transcriptionally active, but may not produce an active virus (Figure 1). The mutant virus proteins produced can be processed and surface displayed, which enhances targeting by HIV-1-specific cytotoxic T lymphocytes (CTLs) (Figure 1) [76]. Alternatively, the uracil containing proviral DNA can result in DNA repair induced degradation (Figure 1) [77]. All of these different fates are also dependent on the extent of Vif-mediated degradation of A3s. 

## 5. The Vif-A3 Interaction and Cross Species Transmission

**Figure 2:**
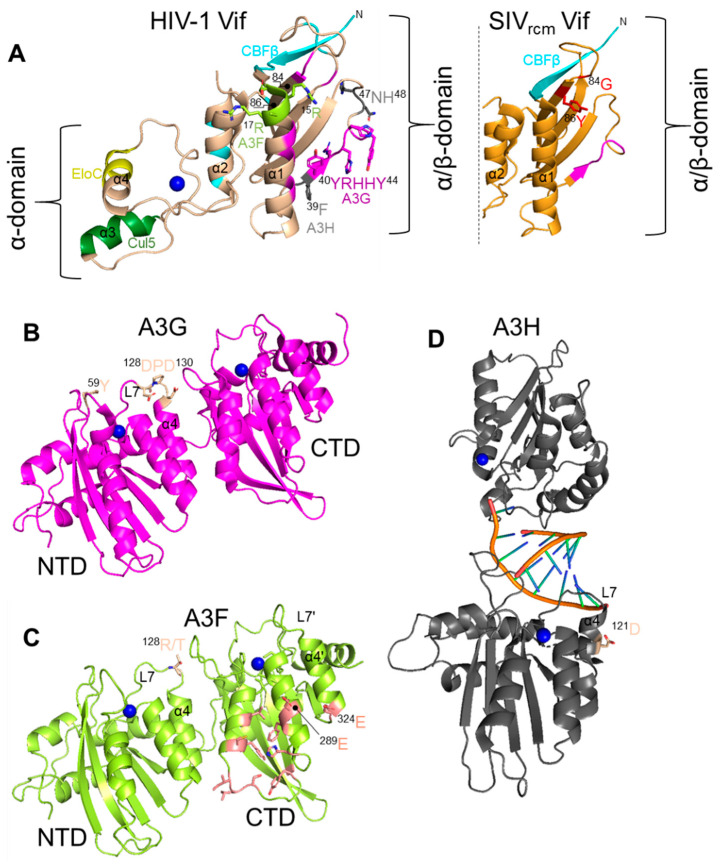
Interaction residues for Vif and APOBEC3s. (**A**) HIV-1 Vif (PDB: 4N9F) has two domains on either side of a bound Zinc (blue). The α-domain contains two alpha helices that mediate two separate interactions with EloC (yellow) and Cul5 (dark green). The N-terminal α/β-domain consists of a five stranded β-sheet, a discontinuous β-strand and three α-helices. The α/β-domain contains the binding interface for CBFβ (cyan) and A3 enzymes. The magenta residues interact with A3G, lime green interact with A3F, and grey interact with A3H. This surface for interacting with A3s is conserved in the hominid lineage. Through mutational and structural studies it was found that OWM additionally use residues 83 to 86. In the SIV_rcm_ Vif structure (partial structure shown on right, PDB: 6P59) residues 83 to 86 are partially on Loop 5, but on HIV-1 Vif they are on a β-strand. The 84 and 86 are key determinants for OWM SIV antagonism of A3F and A3G, respectively. Thus, the OWM SIV Vifs appear to still use the same general interface as hominid SIV/HIV Vifs. (**B**–**D**) A3 enzymes have a basic structure in each ZDD that is composed of a five-stranded β-sheet core surrounded by six α-helices. Zinc atoms are shown as blue spheres. (**B**) Rhesus Macaque A3G (PDB: 6P40) is shown. A3G interacts with Vif on the NTD using three primary amino acids from 128–130 on loop 7 (L7). The human A3G amino acids are denoted in large orange letters. These three amino acids most often differ between species. Amino acid 59 is adjacent to the DPD motif and was found to be polymorphic between Sooty Mangabeys and Rhesus Macaque. (**C**) Model of Rhesus Macaque A3F generated from PDB: 6P40 using SWISS-MODEL [95]. HIV-1 Vif primarily interacts with human A3F on the CTD with residues E289 and E324 identified as major contact points. HIV-2 and SIV_smm_ interact with human/sooty mangabey A3F on the NTD using residue 128. This is analogous to the region Vif interacts with A3G. (**D**) A3H (PDB: 6B0B) is an obligate dimer with dimerization mediated by a double-stranded RNA. The equivalent amino acid to A3G and A3F 128 is A3H 121. In A3H, this amino acid is on α-helix 4 (h4), rather than the adjacent loop 7 (L7). Human A3H Haplotype II is shown. Figures were made using PyMOL (the PyMOL Molecular Graphics System, Version 1.5.05, Shrödinger, LLC, Germany).

All lentiviruses except the equine infectious anemia virus encode a protein Vif during the late phase of replication [78]. Vif interacts with the cellular proteins Cullin 5 and Elongin C in order to be recruited into an E3 polyubiquitin ligase complex [79,80,81,82,83,84,85,86]. All primate lentivirus Vifs also interact with the co-transcription factor CBF-β for stability [87,88,89]. HIV-1 Vif has also been shown to be further stabilized by binding Elongin C (Figure 1) [90]. Vif alone is a highly unstructured protein but when coupled has two main domains, the α-domain and the α/β-domain [86]. The α/β-domain interacts with CBF-β and A3 enzymes and the α-domain interacts with Elongin C and Cullin 5 (Figure 2) [86]. The rest of the protein consists largely of loops and contributes to its thermodynamic instability (Figure 2) [86]. Non-primate lentivirus Vifs have other mechanisms for stability. Bovine immunodeficiency virus Vif has an additional protein domain that is assumed to stabilize the otherwise flexible and largely unstructured Vif and the Sheep Maedi-visna Virus Vif is stabilized by Cycolophillin A [91,92,93]. In addition, the Elongin C interacts with Elongin B and the Cullin 5 interacts with Rbx2 [94]. The final complex in primates contains Vif/CBF-β/Elongin B/Elongin C/Cullin 5/Rbx2 and an E2 ubiquitin ligase (Figure 1). Vif is the substrate receptor that replaces the host SOCS2 protein and recruits and induces Lysine 48 linked polyubiquitination and degradation of A3 enzymes through the proteasome pathway (Figure 1) [79,84,85].

There is also considerable variation in the ability of Vif to induce degradation of A3 enzymes and even in the presence of Vif, some quantities of A3 enzymes are still encapsidated. HIV-1 tries to avoid A3 encapsidation by blocking HIV-1 assembly until Vif expression has peaked and depleted A3 levels [96]. This leads to lower levels of mutagenesis, which some have reported may be beneficial for the virus since it promotes evolution, however, other studies have found even low levels of A3-induced mutagenesis lowers HIV-1 fitness under selective conditions [75,97,98]. Thus, the A3-mediated restriction is a numbers game and restriction in the presence of a Vif adapted to the specific host A3s is not guaranteed, although nearly 30% of nonfunctional but integrated HIV-1 genomes sequenced from HIV-1+ individuals show the characteristic C/G→T/A mutations induced by A3s [99]. 

**Figure 3:**
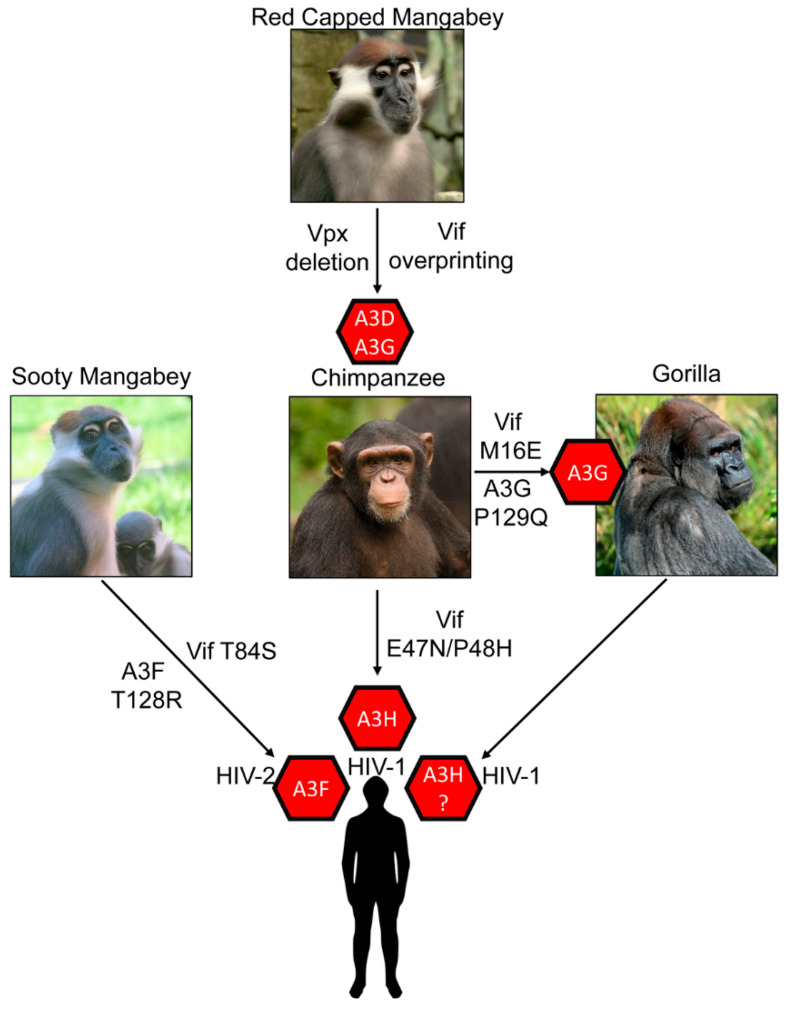
Overview of transmission barriers and associated mutations. The SIV_cpz_ is most similar to a SIV_rcm_–like virus (Red Capped Mangabey), although the *vpu* and *env* genes are more similar to SIV from *Cercopithecus* monkeys. This recombination within chimpanzees created SIV_cpz_ that had a deletion of *vpx* and generated a unique *vif* that overlaps with *vpr* at its 3’ end and contains additional amino acids by a process called overprinting. This new Vif protein could counteract chimpanzee A3D and A3G and facilitated the cross-species transmission. Once in the chimpanzee, the SIV was more easily transmissible to humans and gorilla. Gorilla A3G is resistant to SIV_cpz_ Vif due to a P129Q change from chimpanzee to gorilla A3G. This necessitated a corresponding change in SIV_cpz_ Vif (M16E) to antagonize gorilla A3G and cross the species barrier. The gorilla A3G is polymorphic at amino acid 129 (P/Q) suggesting that this could render certain gorillas more susceptible to transmission of SIV_cpz_ allowing the virus to gain foothold. It is also possible that spread of SIV_gor_ selected for an increased frequency of the A3G 129Q change in gorillas. The SIV_cpz_ and SIV_gor_ were transmitted to humans. For SIV_cpz_, stable A3H haplotypes posed a barrier and forced the evolution of SIV_cpz_ Vif (E47N/P48H) to form HIV-1. Thus, it has been suggested that humans with unstable A3H haplotypes were more susceptible to infection with SIV_cpz_, and possibly the same scenario has occurred for the SIV_gor_ transmission that resulted another HIV-1 group. For the direct transmission of SIV_smm_ to humans, which resulted in HIV-2, recent evidence suggests that A3F posed a transmission barrier. The A3F in sooty mangabeys and humans differ by a T128R change and this required a corresponding change for Vif antagonism (T84S).

The most striking outcome of A3s blocking cross species transmission is the forced evolution of SIV that transmitted from OWM to chimpanzee. For this transmission to occur, there was a prior recombination event in chimpanzees from multiple SIVs from OWMs (from Red-Capped Mangabeys and *Cercopithecus* monkeys) to form SIV_cpz_ (Figure 3) [100]. This evolution is distinctive since it required a complete deletion of OWM SIV *vpx* gene and also resulted in additional amino acids being added to the overlapping region of *vif* that was deleted, an effect called overprinting [100]. The overprinting region of *vif* was essential for successful antagonism of chimpanzee A3s and human A3D, A3F and A3G [100,101].

After this adaptation, the barrier for cross species transmission of SIV_cpz_ to humans was lessened and there are three well documented transmissions resulting in HIV-1 Groups M, N and O. The HIV-1 Group M has nine strains (A-K) and accounts for the majority of infections worldwide [102]. It is known that differences in tetherin antagonism among the HIV groups have determined which HIV-1 lineages have become pandemic or endemic [103]. An equivalent study has not been done focusing on A3 enzyme antagonism. There is also an HIV-1 Group P that resulted from transmission of SIV_cpz_ to gorillas and then SIV from gorillas (SIV_gor_) to humans (Figure 3) [104]. HIV-2 on the other hand was a direct transmission of SIV from Sooty Mangabey (SMM) into humans and resulted in Groups A–I (Figure 3) [1,11]. The HIV-2 lineage is interesting since the SIV_smm_ appears to be preadapted to suppress many human restriction factors, but at the same time HIV-2 is less transmissible and less pathogenic than HIV-1 [102]. Thus, these transmission barriers have forced completely different evolution of the two HIVs.

The Vif-A3 barrier is acting at a protein-protein level with specific amino acid interactions occurring on Vif for each A3 and on each A3 for Vif. These interaction interfaces have been extensively reviewed elsewhere [31,105,106,107] and here we focus on discussing the key amino acids or A3s that have been shown to have acted as a transmission barrier, forcing evolution of the SIV Vif. 

## 6. APOBEC3G

Several studies have shown that a physical interaction of Vif and an A3 is required for Vif-induced degradation to occur. Initial studies reported that the Vif protein of HIV-1 can induce degradation of human and chimpanzee A3G, but cannot antagonize A3G from AGM and Rhesus Macaque [82,108,109,110,111]. Conversely, Vif from SIV_AGM_ can antagonize AGM A3G but is ineffective against human A3G [82,108,109,110,111]. To identify amino acids critical for interaction of HIV-1 Vif and human A3G, the human A3G amino acids were substituted with those from Rhesus Macaque or AGM A3G. Replacement of human A3G 128D with 128K as found in AGM A3G abolished the interaction of HIV-1 Vif with human A3G and hence this mutant A3G was no longer sensitive to HIV-1 Vif mediated degradation [82,108,109,110,111]. However, when the 128D was substituted by 128A, HIV-1 Vif and A3G interaction was intact and Vif could induce degradation of mutant A3G suggesting that electrostatic interactions of amino acids are more important than the identity of amino acids [109,110]. Later studies confirmed the importance of a 128D of A3G and demonstrated that a 129P and 130D also contribute to interaction with Vif (Figure 2) [112,113,114]. In addition to HIV-1, Vifs from HIV-2 and SIV originating from Sooty Mangabey monkeys (SIV_smm_) and Red-Capped Mangabey (RCM) also use this 128–130 region to bind A3G [115,116]. The human A3G 128–130 region was first found to interact with HIV-1 Vif at ^40^YRHHY^44^ [117] (Figure 2). Later, using Vif-mediated degradation assays, patient-derived Vif variants, and HIV-1 forced evolution experiments, it was found that human A3G interacts with HIV-1 Vif at ^40^YRHHY^44^ and ^15^DRMR^17^ [118]. The conserved ^15^DRMR^17^ sequence was first found to interact with human A3F and did not affect A3G degradation using alanine scanning mutagenesis (Figure 2) [117]. Thus, the role of the ^15^DRMR^17^ sequence in binding human A3G may require clarification by structural studies. However, mutating the HIV-1 Vif DRMR region to SEMQ or SERQ as found in SIV_agm_ Vif enhances the interaction of HIV-1 Vif with Rhesus Macaque A3G, AGM A3G and D128K A3G [119]. Thus, there is a precedent for involvement of this region in species other than in humans. These earlier studies based on site directed mutagenesis are supported by crystal structures of Vifs from both HIV-1 and the SIV_rcm_ that showed the proposed residues for interaction are surface exposed [86,116]. The Vif of SIV_rcm_ was shown to interact with the chimpanzee A3G using a ^42^YVPHF^46^ motif that is similar in sequence and hydrophobicity to HIV-1 Vif ^40^YRHHY^44^ [116]. Further, both The SIV_rcm_ and SIV_cpz_ Vifs were found to use Loop 5 residues, ^83^LGTY^86^ in SIV_rcm_ Vif and HLGH in SIV_cpz_ Vif, to antagonize human A3G, with 86Y in SIV_rcm_ Vif being the primary determinant (Figure 2) [116]. The composition of the residues on Loop 5 determined if the Vif had a broad or specific activity toward different A3Gs [116]. Importantly, the structural studies showed two key features of the “arms race” strategy from the virus side, that Vif adaption to bind to human A3s either occurs on loop regions of the protein, which can sustain more changes than structured regions and that since Vif interacts with some A3s on a distributed surface if one surface cannot bind, then other residues can maintain the interaction until evolution finds new contact points (Figure 2) [116]. This has been suggested previously using molecular dynamics modeling of Vif and A3F and termed the “wobble model” of host-pathogen adaptation [120,121]. 

On the host side of the “arms race”, point mutations in primates could not keep up with the viral evolution rate. Thus, the A3 family faced selective pressure from infectious agents and that resulted in duplication events (resulting in seven enzymes) and multiple polymorphisms for each A3 [122,123,124,125,126]. Heterozygosity of a restriction factor can confer selective advantage to the host since it forces the virus to evolve to bind multiple alleles of the host factor. Compton et al. while studying evolution of A3G in OWMs observed that A3G was polymorphic in AGM [127]. They found that some single amino acid changes conferred resistance of AGM A3G against SIV_agm.ver_ (vervet monkey species) and SIV_agm.tan_ (tantalus monkey species) Vifs [127]. In an experimental infection study in AGM cells, they found out that viral adaptation to antagonize A3G was impaired in the virus obtained from monkeys that were heterozygous for A3G, where one allele was resistant to Vif-mediated degradation [127]. However, the SIV from monkeys that had both A3G alleles resistant to Vif-mediated degradation was able to evolve to gain the ability to counteract A3G [127].

Polymorphic forms in A3G have also been found to create a strong barrier to cross-species transmission of SIV_smm_ to SIV_mac_ (Rhesus Macaque). This transmission was of SIV_smm_ in a colony of captive Rhesus Macaques. Although not a natural infection, it still exemplifies how polymorphisms combat viral adaption. Krupp et al. investigated if A3G acted as a cross species transmission barrier for transmission of SIV_smm_ (reservoir host) to a new host, Rhesus Macaques [128]. They found that Rhesus Macaque A3G was resistant to SIV_smm_ Vif and they attributed this resistance to the polymorphism they found in the N-terminal domain (NTD) of Rhesus Macaque A3G [128]. The highly conserved 59Y was found to be replaced by two amino acid insertions, either leucine-leucine (59L/60L) or leucine-arginine (59L/60R) [128]. On the surface of A3G, these residues are in close proximity to Amino Acid 128 and appear to have driven adaptation of SIV_smm_ Vif at Position 17 (glycine to glutamate) in order to induce degradation of Rhesus Macaque A3G and formed SIV_mac_ (Figure 2) [128]. This further exemplifies a common interface on Vif to interact with A3G from multiple species. 

The gorilla A3G has also been found to have been a barrier for infection of SIV_cpz_. Nakano et al. found that gorilla A3G was resistant to degradation by Vif from SIV_cpz_ from *Pan troglodytes troglodytes* and required an M16E mutation to induce degradation of gorilla A3G [129]. The SIV_gor_ Vif has an E16 residue indicating that this adaption was essential in breaching the cross species barrier (Figure 3) [129]. Further, the interaction occurred on gorilla A3G at amino acid Position 129, within the same amino acids that HIV-1 Vif interacts with human A3G (^128^DPD^130^) (Figure 2) [129]. A P129Q change from chimpanzee A3G to gorilla A3G enabled resistance to SIV_cpz_ Vif until the M16E adaptive mutation occurred (Figure 3) [115]. 

## 7. APOBEC3F

There is an abundance of literature on Vif adaptations to A3G since it is in all primates one of the most active A3 enzymes for lentiviral restriction [130]. Human A3F has been characterized as two- to four- fold less restrictive towards HIV-1 ΔVif than A3G, but this is dependent on several factors [131,132]. First, A3F exists in humans as two polymorphic forms, 231I and 231V. These two forms of A3F are equally present in the population and most often occur in individuals as heterozygous alleles [132]. The A3F 231V is more stable in cells and thus more active in restricting HIV-1 ΔVif [132]. Although data has yet to show if these can act as an infection barrier as was shown for AGM A3G polymorphic alleles [127], it is known that the A3F 231I and 231V can form oligomers in cells (a trimer or dimer of A3F) and these oligomeric forms are more resistant to Vif-mediated degradation [132]. In addition, due to the similar amino acid sequences of A3F and A3G, these two A3s can form hetero-oligomers [133]. Interestingly, in this complex, only the A3F, but not the A3G becomes more resistant to Vif-mediated degradation [75]. The mechanism of this resistance is not known. Further, since A3s have largely been studied individually to determine cross-species barriers, it would be interesting to repeat some of the studies with A3G, but co-express A3F and determine if A3F also had a role in establishing a cross species barrier. In human cells, HIV-1 with stop codons introduced into Vif will evolve to induce degradation of A3F, but not A3G [134]. The HIV-1 evolution experiments with A3G did involve HIV-1 suppressing A3G encapsidation by producing more virions, which had a dilution effect since A3 encapsidation is stochastic [135,136]. However, the studies showed that A3F puts considerably more selective pressure on *vif* evolution than A3G, and thus is still important to consider in cross species transmissions [134]. 

A recent study suggests that A3F has posed a cross species transmission barrier for SIV_smm_ transmission to humans. The SIV_smm_ transmission resulted in HIV-2 (Figure 3). It was found that different lineages of SIV_smm_ were capable of counteracting human A3 proteins in a Vif-dependent manner, but not as efficiently as HIV-2, indicating some adaptation to humans occurred [137]. A3F was the most resistant to SIV_smm_ Vif-mediated degradation, suggesting that it posed the greatest barrier to transmission [137]. The SIV_smm_ Vif contains a T84, which is S84 in HIV-2 Vif. Introducing a T84S mutation in SIV_smm_ increased its ability to induce degradation of human A3F (Figure 3) [137]. 

Further, since it had been previously shown that HIV-2 Vif interacts with A3F in the NTD, whereas HIV-1 Vif interacts with A3F in the CTD, mutations were made in A3F at the NTD Position 128, analogous to the same position that many Vifs interact with A3G [138]. Changing the Sooty Mangabey A3F by a T128R mutation to match human A3F increased its activity against SIV_smm_, but not HIV-1 and HIV-2 (Figure 2 and Figure 3) [137]. In support of this being an adaptive mutation, HIV-2 was more sensitive to the R128T mutant form of human A3F [137]. Surprisingly, the R128T change also increased the antiviral activity of human A3F against HIV-1 [137]. 

Previous published reports have shown that HIV-1 Vif interacts with the CTD of A3F with amino acids E289 and E324 being major Vif interaction points across a dispersed interaction interface (Figure 2) [139,140]. It had been proposed that HIV-1 Vif may interact with the N-terminal domain of A3F [139] and Vif interactions with this region of A3G and A3H are well documented [82,109,112,115,119,141], although the interaction in the NTD of A3F was not considered a primary determinant of the Vif interaction. Most recently, a cryo-EM structure of Vif, CBF-β, and the CTD of A3F showed that both Vif and CBF-β interact with the A3F CTD domain by forming a platform [142]. Thus, the interaction of HIV-1 Vif with A3F and the role of A3F in blocking cross-species transmission warrants further study.

## 8. APOBEC3H

Among A3 genes, human A3H is the most evolutionary divergent gene and exists as seven major haplotypes and many splice variants of human A3H have been reported [32,45,143,144]. These polymorphic forms of A3H differ significantly in their stability and several studies have reported that stability of human A3H, which is linked to its cellular localization, is one of the important determinants of its antiretroviral property [49,51,145]. Recently, it was shown that different haplotypes have different levels of polyubiquitination and proteosomal degradation in cells [50]. Further, A3H inherent enzyme stability is determined by its dimerization state [146,147,148]. Dimers are formed with no protein-protein contacts, but are mediated by a double-stranded RNA that imparts stability to A3H and promotes localization in the cytoplasm (Figure 2) [147,148,149,150]. The single nucleotide polymorphisms that combine to form the seven major haplotypes are at four positions, one of which results in a change at Amino Acid 121, the primary Vif interaction site (Figure 2) [45]. The most common circulating A3Hs in the population are unstable and inactive against HIV-1, i.e., Haplotypes I, III, IV, and VI [145]. Since the Vif is always evolving due to pressures to bind other A3s or natural drift, the majority of HIV-1 Vifs cannot induce degradation of the more rare stable A3H Haplotypes II, V, and VII [43,47]. The impact of this was shown experimentally.

In a study examining the impact of A3H polymorphisms in the human population, HIV-1+ participants that had one or two A3H alleles active against HIV-1 (A3H Haplotype II) had lower viral loads early in infection compared to participants with A3H alleles not active against HIV-1 [43]. This is because the HIV-1 Vif had a 39V which is not able to induce degradation of A3H, but can induce degradation of A3F and A3G (Figure 2) [43]. Forced evolution experiments to find Vifs that could induce degradation of A3H resulted in a 39F [43]. Importantly, this 39F correlates with data from circulating HIV-1 genome sequences where HIV-1 from African regions primarily contained a 39F and people of African descent are more likely to carry A3H alleles active against HIV-1 [47]. Conversely, the Vif obtained from HIV-1 circulating in the Americas primarily had a 39V and correlates with populations that primarily carry A3H alleles inactive against HIV-1. Thus, the “arms-race” is on-going with the most polymorphic A3 and has great relevance for HIV-1 infection and progression to AIDS [43,47].

Zhang et al. found that A3H Haplotype II, in addition to A3G, acted as a cross species transmission barrier for SIV_cpz_ to humans [101]. The study found that A3H Haplotype II is resistant to degradation induced by SIV_cpz_ Vif and concomitantly was found to reduce the infectivity of SIV_cpz_ [101]. The chimpanzee A3H was antagonized by SIV and HIV-1 Vifs but human A3H Haplotype II was resistant to Vifs from SIV_cpz_ and SIV_gor_. From their study they speculated that cross species transmission of SIV_cpz_ to humans may have first occurred in humans that expressed an unstable form of A3H protein, e.g., A3H Haplotypes I, III, IV, or VI [101]. SIV_cpz_ and SIV_gor_ Vifs were already effective antagonists of human A3D, A3F and A3G proteins [100,101]. However, human A3H Haplotype II appears to have played a role in limiting the cross-species transmission of SIV_cpz_ from chimpanzee to humans. This could also be true for SIV_gor_ as well (Figure 3). Zhang et al. revealed that two amino acids substitutions in Vif (E47N/P48H) enabled SIV_cpz_ Vif to induce degradation of human A3H (Figure 2 and Figure 3) [101].

## 9. Conclusions

Several common interaction interfaces on both Vif and A3 enzymes have been identified. This is evolutionarily favorable since the interaction region can readily evolve without destabilizing the protein (Figure 2 and Figure 3). Mutations in loop regions or a surface exposed region can enable toggling between positive and negatively charged amino acids (Figure 2). For A3G and A3H, the interaction occurs on analogous regions, Residues 128–130 in A3G and Residue 121 in A3H (Figure 2). The human A3F has major interactions in the CTD (Figure 2). The corresponding interaction in HIV-1 Vif is primarily residues 15-17 for A3F, 39, 47 and 48 for A3H, and both 15–17 and 40–44 for A3G (Figure 2). The SIV_gor_ Vif interaction site for gorilla A3G also overlaps with HIV-1 Vif interaction site and is at position 16. However, for OWMs, their Vif interaction sites tended to cluster differently on Vif and were at Residue 84 for SMM and Residue 86 for RCM, although this is near Residues 15–17 (Figure 2). The OWM A3s still appear to interact at the 128 to 130 region. Thus, HIV-1 Vif that interacts with A3F, A3D, and A3C in the C-terminal domain on a diffuse interaction site involving 11 amino acids from Position 255 to 324 is unique (Figure 2) [151]. These common interfaces support the wobble hypothesis in that there are small changes being made over time in a specific area, rather than large changes in the interfaces to block an interaction or adapt to a new interaction [120]. Since the interaction strength of the Vif and A3 determines the degradation efficiency [152], only small changes would be needed to reestablish a robust interaction.

It has been suggested that inefficient interactions of Vif with A3s may actually be beneficial to the virus. During HIV-1 infection, *vif* can become mutated to produce a protein that does not efficiently induce degradation of A3s [153]. This allows for more A3 encapsidation and more mutations to occur. However, since the mutations are stochastic, there is no definitive statement that can be made regarding the outcome. Some studies have found that this accelerates drug resistance to a single antiretroviral drug, but another study that used multiple drugs as in antiretroviral therapy found that A3-mediated mutagenesis actually decreased HIV-1 fitness and that reverse transcriptase alone was still more likely to produce drug resistance mutations [75,97,98,154]. With immune escape, similarly divergent results have been found where deamination in some epitopes can increase recognition of HIV-1 by immune cells, but other epitopes facilitate HIV-1 immune escape [76,155,156]. In addition, many studies sequenced integrated proviral DNA, which may not produce functional virus and it is unlikely that drug resistant non-functional virus can recombine with other functional viral genomes [157]. However, it appears that once in a host, the relationship between Vif and A3s continues to evolve and is multifactorial. For example, less fit viruses may more easily escape the immune system due to slower replication [158,159]. Thus, there is still more to learn on this topic. 

Future work in the area of A3s and transmission barriers would be interesting to explore with other viruses. Recently A3B has been found to restrict certain herpesviruses and be antagonized by herpesvirus proteins [33]. There may also be A3-mediated restriction of RNA viruses, such as coronaviruses [160,161,162,163]. Regarding current viruses and cross-species transmissions, bats are quite central, and interestingly, they contain 18 different A3s—more than any other mammal [164]. A3s are often called a double-edged sword since they can restrict viruses, but if they are expressed at the wrong time or place, they can induce mutations in host genomic DNA and contribute to cancer evolution [165]. A3B is the best example of the double-edged sword since it has both functions, but otherwise this refers to the family as a whole. A3A and A3H Haplotype I are the other A3s involved in cancer and they are not known to restrict many viral pathogens, but can restrict retroelements [34,166,167]. Thus, if A3s have a role in suppression of bat viruses, it would be very pertinent to determine if they also become a double-edged sword and could facilitate viral evolution and cross-species transmission out of bats to other organisms. 

In summary, A3 enzymes shaped the evolution of SIV and HIV through the ongoing suppression and avoidance related to Vif. As we learn of more viruses that are restricted by A3s in a deamination-dependent manner it would be interesting to realize if there are parallels with the SIV and HIV paradigm or new ones to be discovered. There is still much to learn about A3s and lentiviruses as we discover more A3 polymorphisms, ability of A3s to hetero-oligomerize, and the fate of A3-induced mutations in an organism over the lifetime of an infection.

## Data Availability

Data is available in the referenced literature.
